# Controllable size-selective method to prepare graphene quantum dots from graphene oxide

**DOI:** 10.1186/s11671-015-0783-9

**Published:** 2015-02-08

**Authors:** Tianju Fan, Wenjin Zeng, Wei Tang, Chunqiu Yuan, Songzhao Tong, Kaiyu Cai, Yidong Liu, Wei Huang, Yong Min, Arthur J Epstein

**Affiliations:** Institute of Advanced Materials, Nanjing University of Posts and Telecommunications, 9 Wenyuan Road, Nanjing, Jiangsu 210046 China; State Key Laboratory of Organic Electronics and Information Displays and Fountain Global Photoelectric Technology Co. Ltd, 2 Xinyue Road, Yancheng, Jiangsu 224000 China; Department of Physics and Chemistry & Biochemistry, The Ohio State University, Columbus, OH 43210 USA

**Keywords:** Graphene oxide, Graphene quantum dots, Chemical cutting, Fluorescence mechanism, 81.07.Ta (fabrication of quantum dots), 68.65.Hb (structure and nonelectronic properties of quantum dots), 61.48.Gh (graphene structure)

## Abstract

We demonstrated one-step method to fabricate two different sizes of graphene quantum dots (GQDs) through chemical cutting from graphene oxide (GO), which had many advantages in terms of simple process, low cost, and large scale in manufacturing with higher production yield comparing to the reported methods. Several analytical methods were employed to characterize the composition and morphology of the resultants. Bright blue luminescent GQDs were obtained with a produced yield as high as 34.8%. Moreover, how the different sizes affect fluorescence wavelength mechanism was investigated in details.

## Background

The unique structure of monolayered graphene was composed of a one-atom-thick two-dimensional crystal of sp^2^ carbon atoms arranged in a honeycomb lattice, which had been attributed to its extraordinary electronic properties such as high intrinsic mobility and excellent thermal and electrical conductivities. Those characteristics of graphene had led to extensive applications in the fields of electronic devices, including photovoltaic cells, supercapacitors, and flexible touch screens [1-3]. Regarding to the superlative electronic properties, recent studies have demonstrated the graphene’s photoluminescent characteristics, which could expand its application to optical-related fields [4,5]. The photoluminescence (PL) property of graphene was derived by controlling the zero-band gap of graphene [6]. Since the bandgap could be tuned by the size, shape, and fraction of the sp^2^ domains in the sp^3^ matrix, a variety of graphene derivatives were explored as PL graphene moieties [7,8]. As an initial endeavor, the optical property of graphene oxide (GO), which was chemically prepared using the modified Hummers method from graphite powders, was investigated [9,10]. The produced GO, which ranged from a few tens of nanometers to several tens of micrometers in the lateral dimension, contained various shapes and nanosized sp^2^ carbon domains localized by the sp^3^ carbon structures, resulting in semiconductive and PL properties [11]. In addition to the control of sp^2^ carbon domain by reduction process, several reports have focused on modulating the size of graphene itself to less than 100 nm, graphene quantum dots (GQDs), to endow quantum confinement effect that was also a contributing factor for PL emission [3]. Such GQDs display moderate PL signal, non-toxicity, and cell permeability, so their biological applications on cellular imaging, biosensors, and drug delivery were very promising [12]. It was possible to synthesize a nanosized graphene by physical and/or chemical methods, including nanopatterning of graphene by electron beam lithography [13], cage opening of fullerene [14], breakage of GO from micro- into nanosize by hydrothermal and electrochemical methods [15,16], strong acid assisted the cleavage of graphite nanomaterial (nanographite, carbon fibers) [17,18] organic synthesis using polyphenylene dendritic precursors [19], and self-assembly of hexaperihexa benzocoronene [20]. Despite some successes in GQDs synthesis, these methodologies have limitations to overcome. However, the solution-based cleavage method requires harsh oxidation conditions using strong acid and high temperature for a prolonged time, and the organic synthetic methods need a complicated synthetic scheme. The produced GQDs mainly exist as bi- or multi-layers, and the heterogeneity in size and shape was unavoidable. From previous results, we notice that the carbon precursor was an important factor to predetermine the shape and size as well as the exfoliation yield of graphene sheets from graphite sources.

Herein, we employed GO as a starting material to up-down method and synthesized two kinds of GQDs by KMnO_4_/H_2_SO_4_ in oxidized shearing method that had different circular shapes and diameters of less than 10 nm. One kind of GQDs obtained with a shorter reaction time was marked as GQD-1 and gave green PL emitting; the other with longer reaction time was marked as GQDs-2 and gave blue emitting. Their optical properties were compared by means of PL, UV–vis absorbance, to understand the PL mechanism of the nanosized graphene materials. Our proposed methodology allows the production of homogeneous photoluminescent GQDs-1 and GQDs-2 with high yield and high reproducibility.

## Methods

### Chemicals and materials

Graphite powder (200 mesh) was purchased from Baichuan Graphite Ltd. (Qingdao, China). Sulfuric acid (H_2_SO_4_), potassium permanganate (KMnO_4_), hydrogen peroxide (H_2_O_2_), hydrochloric acid (HCl), and sodium hydrate (NaOH) were purchased from Sinopharm Chemical Reagent Co., Ltd. (Shanghai, China). Unless otherwise specified, all reagents were used without further purification.

### Preparation of GO

GO was prepared by the modified Hummers method [21]. Briefly, 1.0 g of graphite and 60 mL H_2_SO_4_ (98%) was stirred in an ice bath, and 5.8 g KMnO_4_ was slowly added with stirring for 0.5 h. The solution was heated to 30°C for 2 h, 40 mL of deionizer water was added slowly, the reaction was heated to 90°C for 30 min, then 80 mL of deionizer water was added. When the temperature was cooled down to 60°C, 10 mL H_2_O_2_ (30%) was added to give an orange yellow solution. Two hundred milliliters of 5% HCl solution was added, the supernatant was decanted and centrifuged with deionizer water to pH 4 to 6, and the mixture solution was further dialyzed in a dialysis bag for 2 days; low density graphene oxide was obtained by lyophilizing at −48°C, 21 Pa, and GO was obtained as gray-yellow powder.

### Preparation of GQDs-1

We suspended GO (1.0 g) in concentrated H_2_SO_4_ for a period of 1 to 2 h in an ice-water bath and then treated them with 50 wt% KMnO_4_. The H_2_SO_4_ conditions aid in shearing the graphene oxide. The reaction mixture was stirred at room temperature for 2 h and then heated to 45 to 50°C for additional 1 h. Distilled water (40 mL) was slowly dropped into the resulting solution. Finally, the reaction temperature was rapidly increased to 90°C with effervescence for 30 min. When all of the KMnO_4_ had been consumed, we quenched the reaction by pouring over ice containing a small amount of H_2_O_2_ followed by distilled water (70 mL) obtaining a yellow transparent solution instantly. After cooling down to room temperature, the mixture was ultra-sonicated mildly for a few minutes, the pH was tuned to 8.0 by NaOH in an ice bath, and we found a black flocculent deposit. Then the pH increases to 4.0 by HCl. The suspension was filtered through a 0.22-μm microporous membrane to remove the large tracts of GO, and deep yellow solution (yield ca. 36%) was separated. The mixture solution was further dialyzed in a dialysis bag (retained molecular weight: 3,000 to 8,000 Da), and greenish fluorescent GQDs-1 were obtained (yield ca. 34.8%).

### Preparation of GQDs-2

We suspended GO in concentrated sulphuric acid for a period of 6 h and then treated them with 60 wt% KMnO_4_. The H_2_SO_4_ conditions aid in exfoliating the graphite oxide and the subsequent graphene structures. The reaction mixture was stirred at room temperature for 1 h and then heated to 55-70°C for additional 1 h. When all of the KMnO_4_ had been consumed by pouring over ice containing a small amount of H_2_O_2_ in the reaction mixture, the solution was filtered over polytetrafluoroethylene (0.22 μm) membrane, and GQDs-2 was obtained by similar method as above.

### Characterization

High-resolution transmission electron microscopic (HRTEM) images were obtained for all samples using a JEOLTEM-2100 F microscope at an accelerating voltage of 200 kV. AFM images were obtained at Brucker A8 instrument. UV–vis absorption and PL spectra were recorded on a Shimadzu UV 3600 spectrophotometer and Shimadzu RF-5301 PC luminescence spectrometer, respectively. While the initial changes in the surface chemical bonding as well as the covalent grafting behavior of the hybrids were recorded by Fourier transform infrared spectrophotometry (FT-IR, IR Prestige 21) in the frequency range of 4,000 to 500 cm^−1^. Raman spectroscopy was performed with JY HR800 micro-Raman system using a 488-nm excitation laser operated at a low power level in order to avoid any heating effect.

## Results and discussion

### The mechanism of GQDs-1 and GQDs-2 analysis

**Figure 1:**
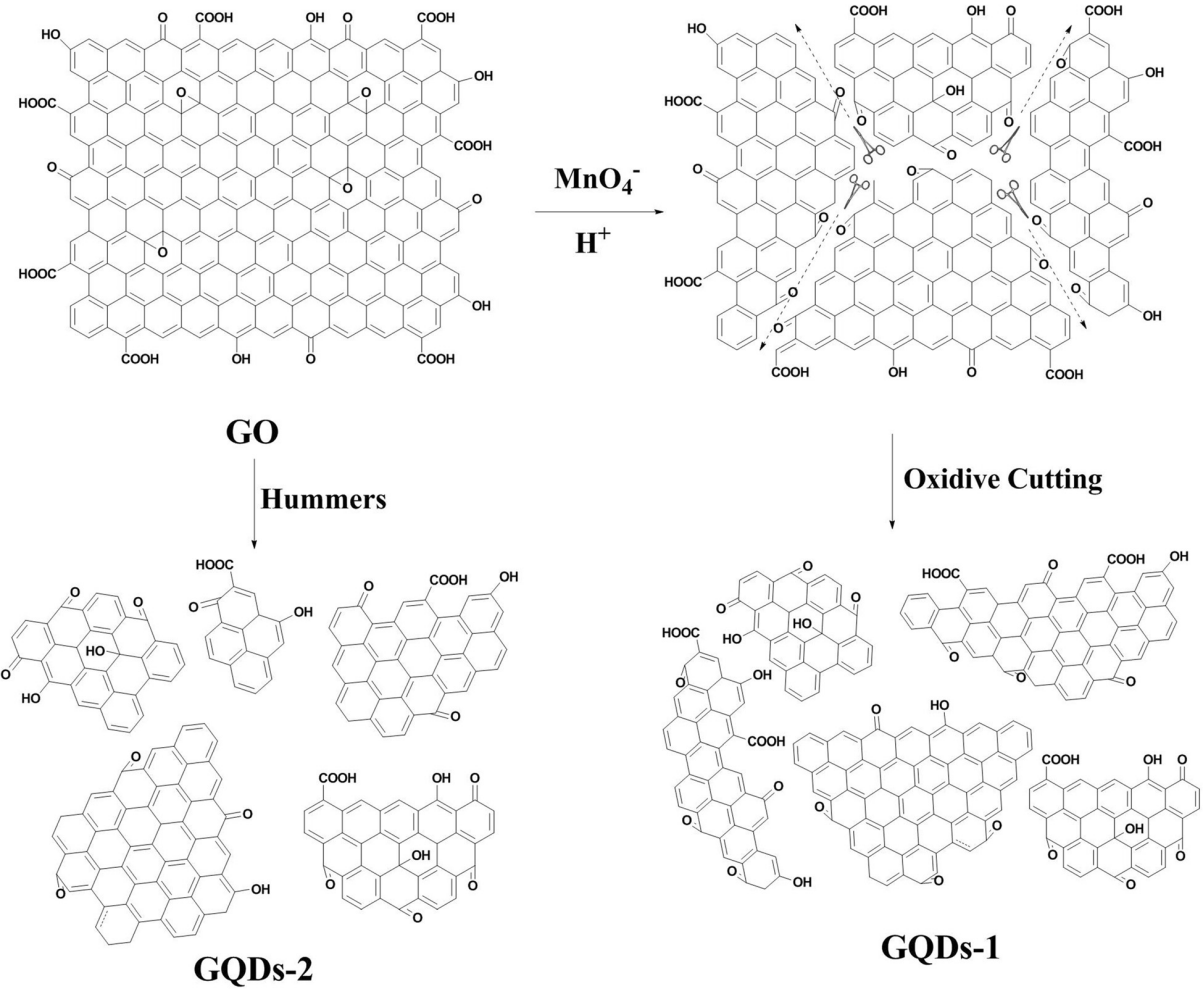
**Mechanism of fabricated GQDs-1 and GQDs-2 for cutting GO by modified Hummers method.**

**Figure 2:**
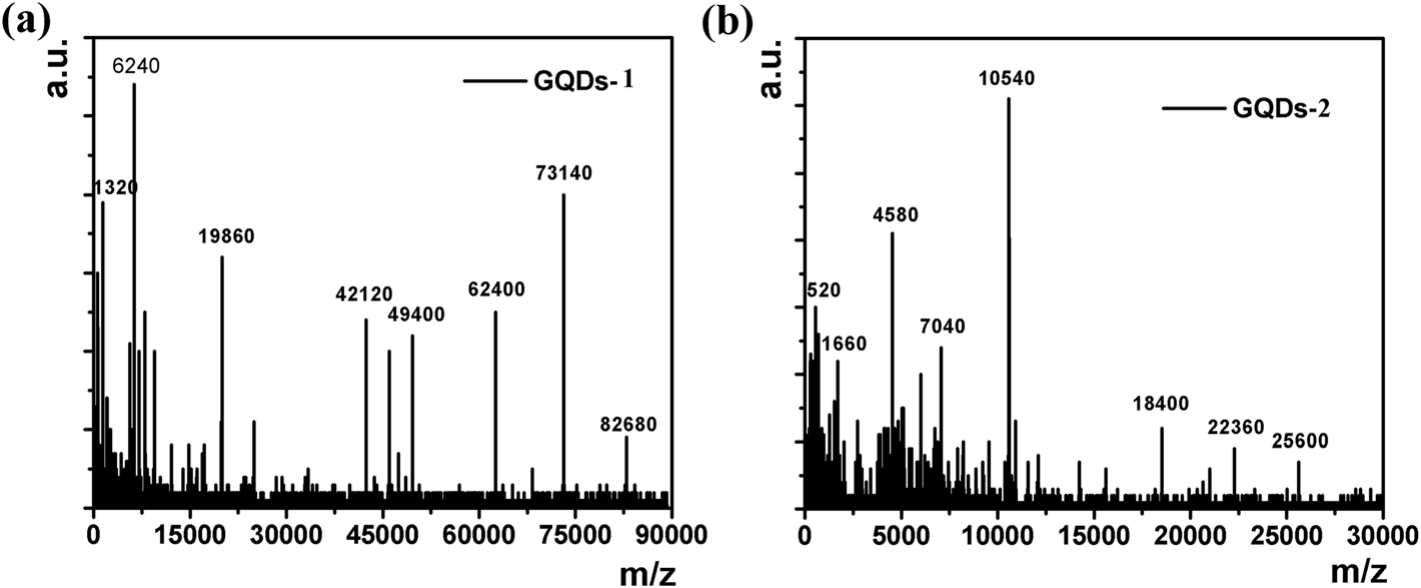
**Preliminary molecular weight of GQDs-1 (a) and GQDs-2 (b) analyzed by MALDI-TOF spectrum.**

Tour et.al [22] used longitudinal unzipping from carbon nanotubes to form graphene nanoribbons with a deep oxidation cutting by KMnO_4_/H_2_SO_4_. However, the mechanism of forming GQDs-1 and GQDs-2 could be explained by the oxidation of alkenes by permanganate in acid as shown in Figure 1. The proposed first step in the process was manganate ester formation as the rate-determining step, and further oxidation was possible to afford the dione in the dehydrating medium position of the buttressing ketones to distort the b, c-alkenes, making them more prone to the next attack by permanganate. As the process continues, the buttressing-induced strain on the b, c-alkenes lessening moreover released more space for carbonyl projection. Herein, the bond angle strain induced by the enlarging hole would make the b, c-alkenes increasingly reactive. Hence, once an opening had been initiated, its further opening was enhanced relative to an unopened graphene oxide sheets in different directions, which attack adjoining C = C/C-C bond. In this case, forming 1,2-diketone from oxidation tailoring in graphene oxide will be in different directions. The ketones could be further converted, through their O-protonated formation, the carboxylic acids that will form large scale of the edges of GQDs. In addition, GQDs-1 was similar as GQDs-2 that fabricated by modified Hummers method. With a different oxidation extent, different sizes of GQDs were obtained. MALDI-TOF spectra demonstrated the weight of molecule of GQDs-1 and GQDs-2 as shown in Figure 2a, b; we could see that the molecule weight of GQDs-1 was about 20,000, while the molecule weight of GQDs-2 was about 80,000. Of course, the MALDI-TOF presumed the GQDs’ molecule weight was imprecise.

### TEM and AFM characterization

**Figure 3:**
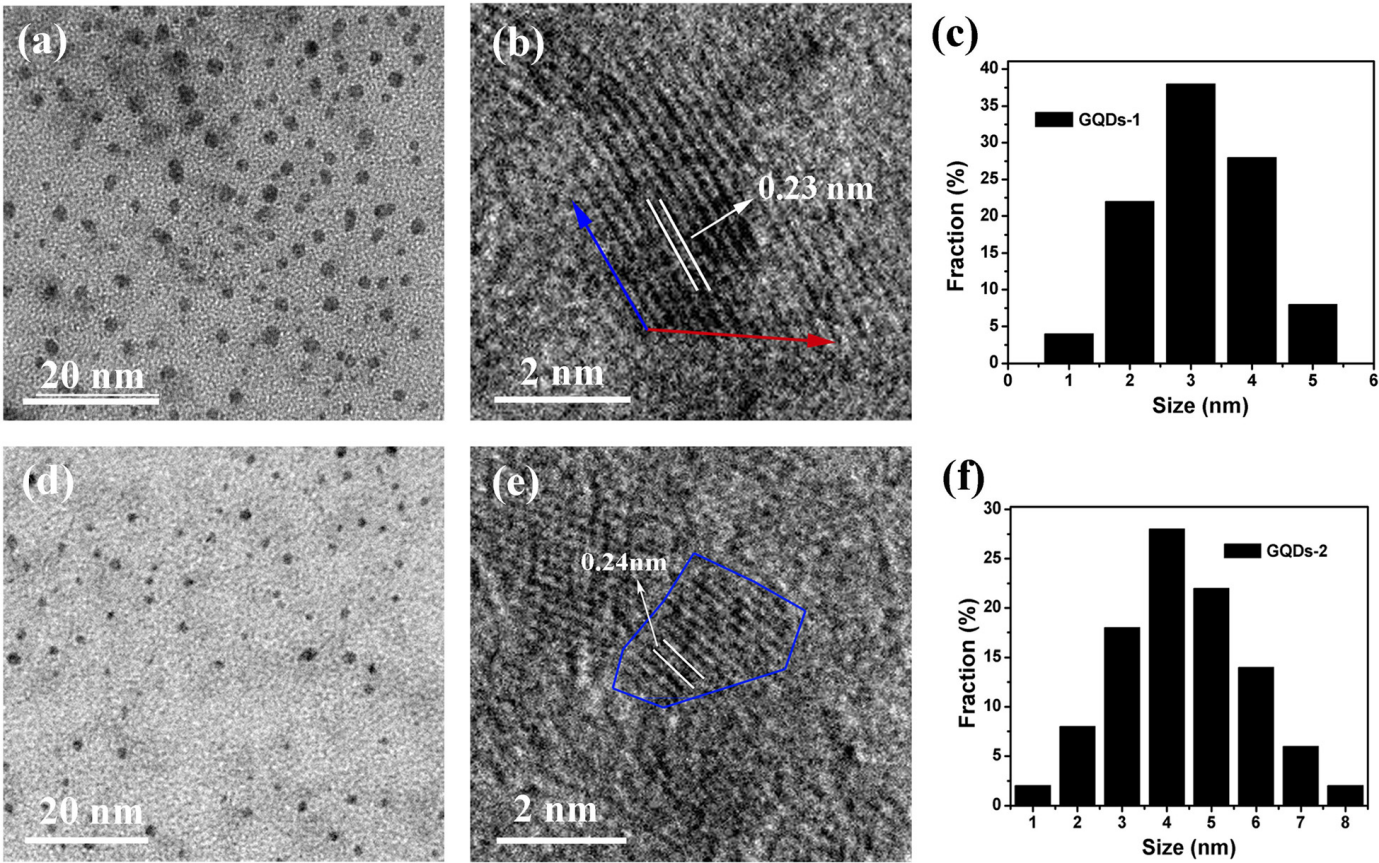
**TEM and AFM characterization. (a)** TEM images of GQDs-1 at 20 nm. **(b)** The HRTEM images of GQDs-1 with measured lattice spacing and edge structures at 2 nm. **(c)** Diameter distribution of the GQDs-1. **(d)** TEM images of GQDs-2 at 20 nm. **(e)** The HRTEM images of GQDs-2 with measured lattice spacing and edge structures at 2 nm. **(f)** Diameter distribution of the GQDs-2.

TEM showed that the collected GQDs-1 was monodisperse and had a uniform diameter of 2–4 nm in size, and GQDs-2 was disperse and had a diameter of 3–5 nm (Figure 3a, d), which was much smaller than those of GQDs (around 10 nm) were synthesized by cutting GO sheets with modified Hummers oxidation cutting methods. In HRTEM images of GQDs-1 with measured lattice spacing, it was shown that GQDs-1 has lattice spacing of 0.23 nm and GQDs-2 has lattice spacing of 0.24 nm as shown in Figure 3b, e. The detailed analysis of HRTEM images for GQDs-1 showed that the periphery of GQDs-1 and GQDs-2 consisted of mixed zigzag and armchair edges. We find that both edges were mixed in curved periphery while the armchair edge appears more frequently in straight periphery. As a consequence, it appeared that armchair edges GQDs-1 consist of both edges and GQDs-2 consist mostly of curved periphery.

**Figure 4:**
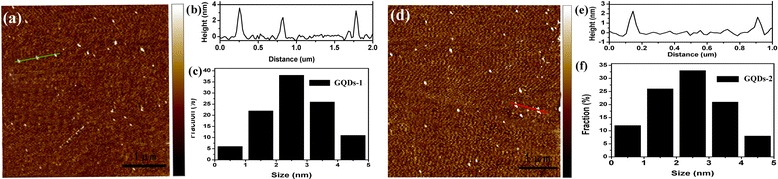
**Images of the morphology of GQDs-1 and GQDs-2 by AFM. (a)** AFM images of GQDs-1 at the range of 5 μm. **(b)** Height profile and **(c)** height distribution of GQDs-1. **(d)** AFM images of GQDs-2 at the range of 5 μm. **(e)** Height profile and **(f)** height distribution of GQDs-2.

Figure 4a, d showed those images of the morphology of GQDs-1 and GQDs-2 by atomic force microscopy (AFM). The heights of GQDs-1 were mainly distributed in the range of 2 to 4 nm with average heights of 2.4 nm as shown in Figure 4b, e, which was similar to previous reports [23,24]. However, the topographic heights of GQDs-2 were mostly between 1 and 2 nm with an average height of 1.6 nm, suggesting that most of GQDs-2 were about five layers as shown in Figure 4c, f. The dimensions and height of GQDs-2 showed no perceptible change, indicating that the PL blue shift of GQDs-2 could be attributed to their size change rather than their dimension variation. Considering the theoretical thickness of a graphene layer of 0.34 nm, the AFM data implied that about five to seven layers of monolayer nanographene sheets consisted of GQDs-1 and GQDs-2.

### FT-IR and Raman analysis

**Figure 5:**
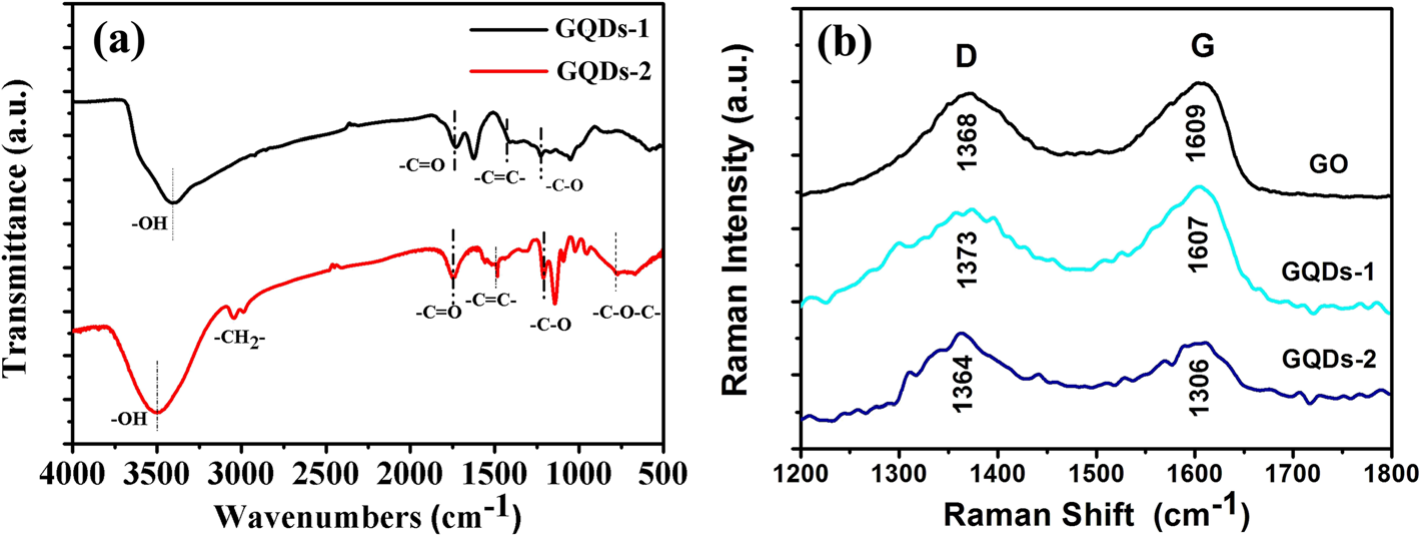
**FTIR and Raman spectroscopy of GQDs-1 and GQDs-2. (a)** FT-IR spectra of GQDs-1 and GQDs-2. **(b)** Raman spectra of GO, GQDs-1, and GQDs-2.

FT-IR was used to characterize the obtained GQDs-1 and GQDs-2 in Figure 5a. Both the GQDs-1 exhibited absorption of carboxyl group and hydroxyl group, showing that both of these two nanomaterials contain –C = O groups. Additionally, the GQDs-1 showed absorption of stretching vibration C-H at 2,950 cm^−1^ and stretching vibration of –C–O–C in the range of below 1,250 cm^−1^. Contrarily, the GQDs-2 exhibited nearly no absorption of –C–O–C, implying that the GQD sample has been reduced partially. Furthermore, the C–OH group (stretching vibration at 1,115 cm^−1^) in the GO had been changed to the C–O–C group (stretching vibration at 1,012 cm^−1^). GQDs-1 noted the characteristic absorption bands of aromatic compound (stretching vibration of C–H in aromatic rings around 3,000 to 3,100 cm^−1^; skeletal vibration of aromatic rings around 1,450 to 1,650 cm^−1^) was included in these FT-IR spectra. However, GQDs showed no aromatic group. After a deep oxidation treatment, the strongest vibrational absorption band of C = O/COOH at 1,724 cm^−1^ became stronger and the vibration band of epoxy groups at 1,052 cm^−1^ almost disappeared.

Figure 5b showed the Raman spectrum of the GQDs-1 and GQDs-2. GQDs-1’s G band (1,611 cm^−1^) and D band (1,367 cm^−1^) were observed with a large intensity ratio *I*_D_/*I*_G_ at 0.99. And GQDs-2’s G band (1,598 cm^−1^) and D band (1,372 cm^−1^) were observed with an intensity ratio *I*_D_/*I*_G_ at 0.97. Raman spectroscopy confirms the quality of the as-prepared GQDs-1 and GQDs-2. The major Raman features were the D band at around 1,367 cm^−1^ and the G band at around 1,608 cm^−1^. Unlike the GQDs-2 synthesized hydrothermally with a much more intense D band, the relative intensity of the ‘disorder’ D band and the crystalline G band (*I*_D_/*I*_G_) for as-produced GQDs-1 and GQDs-2 was only around 0.5, which was similar to that of high-quality few-layer graphene nanoribbons [16]. This indicated not only the high quality of as-prepared GQDs but also the uniqueness of the deep oxidation comparing to the wet chemical method developed here for GQD preparation [25].

### XPS analysis

**Figure 6:**
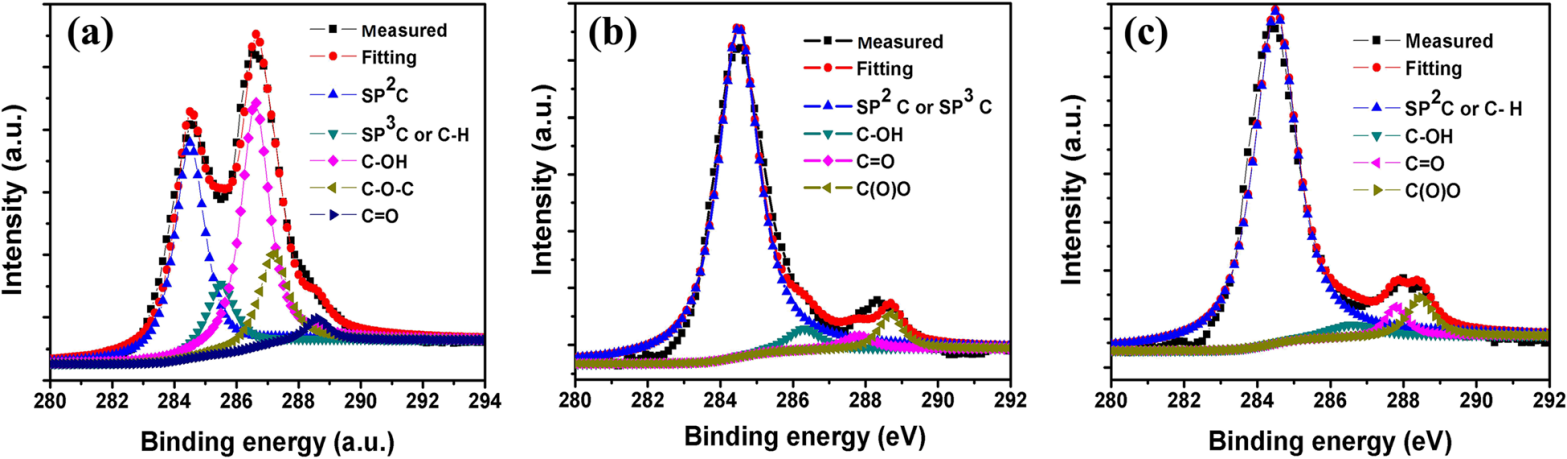
**XPS spectra of GO, GQDs-1, and GQDs-2. (a)** C1s profile of GO, **(b)** C1s profile of GQDs-1, and **(c)** C1s profile of GQDs-2.

To further confirm the functional groups on the surface of the as-prepared GQDs-1 and GQDs-2, X-ray photoelectron spectroscopy (XPS) characterization was carried out. Figure 6a showed the XPS spectrum of C1s of GO as preparation GQDs precursor. The measured spectrum can be deconvoluted into five surface components, corresponding to sp^2^ (C = C) at a binding energy of 284.5 eV, sp^3^ (C–C and C–H) at 285.5 eV, C–OH at 286.8 eV, C–O–C at 287.2 eV as well as C = O or COOH at 288.4 eV. It should be noted that the XPS data presented here represent the surface components of the GO. The surface components of the GQDs as determined by the XPS data were in good agreement with FT-IR results. Figure 6b, c showed the XPS spectra of GQDs-1 and GQDs-2. The GQDs-1 spectrum showed peaks at 284.5, 286.3, 287.8, and 288.4 eV, which corresponded to SP^2^C (C = C) or SP^3^C (C–C, C–H), C–OH, C = O, and COOH chemical binding states, respectively. The XPS spectra of C = O and COOH peaks of GQDs-1 related to GQDs-2 become visibly weakened. This indicated the occurrence of an acid oxidation by H_2_SO_4_/KMnO_4_ second oxidation mechanism that helped to cut the GO into the containing C = O of the edge of GQDs-2. The signal of GQDs-2 at 284 eV assigned to carboxyl groups became weak, whereas the sp^2^ carbon peak at 284.5 eV was almost unchanged. The deoxidization was further confirmed by the changes in the FT-IR and C1s XPS spectra.

The carbon sp^2^ fraction can be evaluated by taking the ratio of the integrated peak areas corresponding to the C–C or C = C peak to the total area under the C1s spectrum from the XPS spectra. And the sp^2^ fractions of GQDs-1 and GQDs-2 were 76% and 86%, respectively. According to Joung’s relationship of the size of the GQDs plotted versus carbon sp^2^ fraction [26], the size of GQDs-1 is about 3.5 nm and that of GQDs-2 is about 4.6 nm which matched our TEM and AFM results.

### UV/Vis and PL analysis

**Figure 7:**
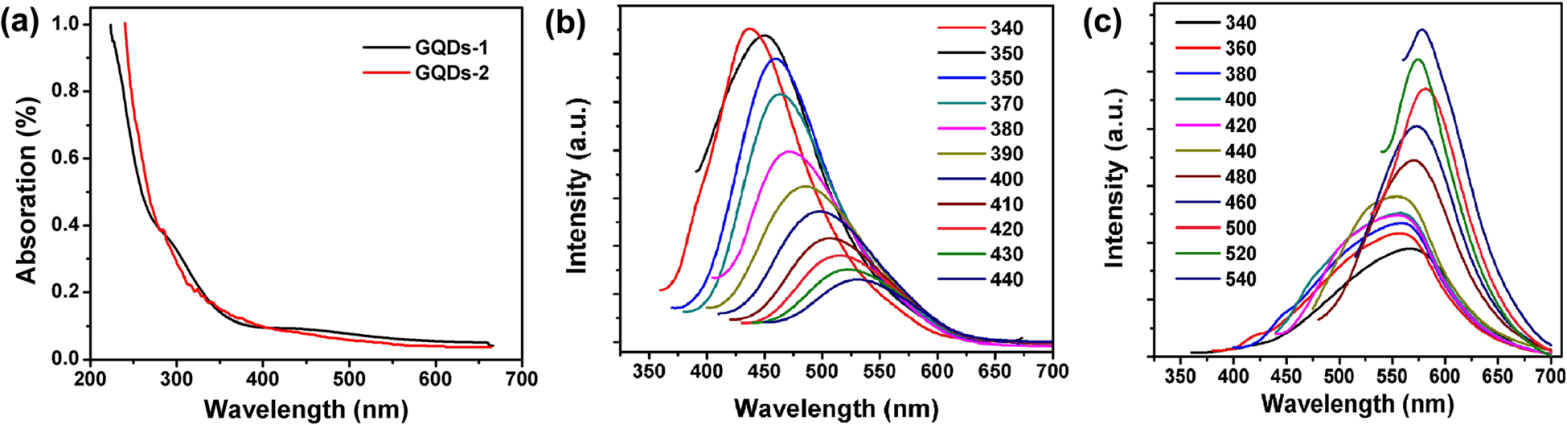
**UV absorption, PL spectrum, and PL property of GQDs-1 and GQDs-2. (a)** UV/Vis absorption of the GQDs-1 and GQDs-2 were dispersed in water; **(b)** PL spectra of the GQDs-1 at different excitation wavelengths from 340 to 440 nm. **(c)** PL spectra of the GQDs-2 at different excitation wavelengths from 340 to 540 nm in water solution.

The UV absorption of the GQDs-1 and the GQDs-2 dispersed in water (Figure 7a). For the GQDs-1, a typical absorption peak at ca. 280, 320 nm was observed, which was assigned to the π-π^*^ transition of aromatic sp^2^ domains [27]. For the GQDs-1, it showed the two sharp peaks at 285 and 325 nm. However, besides the strong π-π* absorption peak at 285 nm, a new absorption band at 325 nm was also observed. Excitation-dependent PL behaviors were common in fluorescent carbon materials.

The PL spectrum of GQDs-1 showed a strong peak with an emission of 476 nm at an excitation wavelength of 340 nm and a weak wavelength with an emission of 560 nm at an excitation of 440 nm with a Stokes shift of 84 nm (Figure 7b). The PL quantum yield of GQDs-1 measured using quinine sulfate as a reference is 7.8%. The PL property of GQDs-1 was attributed to defect state emission into intrinsic state emission. The PL property was possibly attributed to the two or multiphoton active processes [28,29]. The small sizes of GQDs-2 demonstrated the PL property in Figure 7c. The initial GQDs-2 shows a weak wide peak at 526 nm (green emission) when excited under a 340-nm wavelength, and it showed a strong peak at an emission wavelength of 580 nm under an excitation of 540 nm. With a red shift of the excitation wavelength, the emission wavelengths were also redshifted. The emission wavelengths redshift when changing excitation wavelengths from 340 to 540 nm. The PL quantum yield of GQDs-2 measured using quinine sulfate as a reference is 8.9%, which is similar to those reported for luminescent carbon nanoparticles [30,31]. Like most luminescent carbon nanoparticles, the GQDs-1 also exhibited an excitation-dependent PL behavior. When the excitation wavelength was changed from 340 to 440 nm, the PL peak shifts to longer wavelengths and its intensity decreases rapidly, strongest peak was excited at the absorption band. Different scale-sized GQDs were revealed to this fluorescence mechanism; we speculated that the blue emission of GQDs-1 was attributed to electronhole recombination or quantum size effect/zigzag effect (intrinsic state emission).

**Figure 8:**
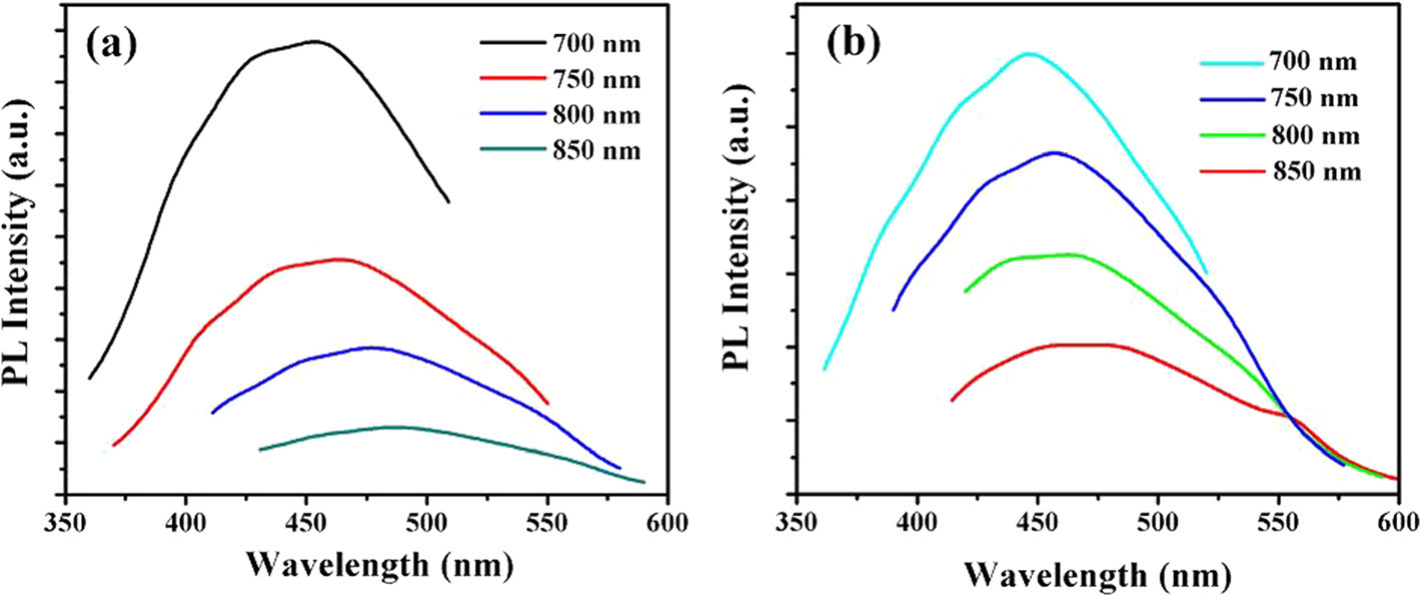
**Emission wavelengths redshift upon changing excitation wavelengths. (a)** Upconverted PL spectrum of GQDs-1 and GQDs-2 **(b)** with excitation at different wavelengths.

With further PL experiment, apart from the strong PL feature, the GQDs-1 and GQDs-2 showed upconversion PL properties. In the upconversion PL behaviors of GQDs-1, GQDs-2 were similar to the downconversion excitation-dependent PL behaviors of their counterparts. In Figure 8a, b, the emission wavelengths redshift when changing excitation wavelengths from 700 to 850 nm. The peak of GQDs-1 was at 458 nm with excitation wavelength is at 700 nm, which was redshift 488 nm with excitation at 850 nm, and the upconverted PL properties of GQDs-2 was similar to GQDs-1. The upconversion PL property of GQDs was possibly attributed to the two or multiphoton active processes. The upconversion PL mechanism in GQDs was still not entirely understood, and other processes such as multiphoton excitation may also be responsible for the observation [32].

## Conclusions

We demonstrated a one-step method to fabricate two different sizes of GQDs-1 and GQDs-2 from GO, which was presented to tune the different sizes of graphene oxide quantum dots at ranges 2 to 4 and 3 to 5 nm and simultaneously tailor its edge structure by oxidation cutting. The resulting GQDs-1 and GQDs-2 thus possessed a photoluminescent property and exhibit a size-dependent shift of emission, respectively. It exhibited that blue luminescent GQDs-1 and green luminescent GQDs-2 were obtained with a produced yield as high as 34.8%. It was shown that the PL property and upconverted PL property of the blue emission of GQDs-1 was attributed to the defect state emission into intrinsic state emission and GQDs-2 was attributed to electronhole recombination or quantum size effect/zigzag effect. The discovery of the GQDs may expand the application of graphene-based materials to other fields such as optoelectronics and biological labeling.

## Abbreviations

GQDs: graphene quantum dots
GO: graphene oxide
PL: photoluminescence
HRTEM: high-resolution transmission electron microscopy
FT-IR: Fourier transform infrared spectrophotometry
AFM: atomic force microscopy
XPS: X-ray photoelectron spectroscopy
